# Association of Life’s Essential 8 with all-cause mortality in asthma patients: evidence from NHANES 2005–2018

**DOI:** 10.3389/fnut.2025.1603875

**Published:** 2025-06-17

**Authors:** Jinqi Zhu, Ran Tao, Sue Zhao

**Affiliations:** ^1^Department of Respiratory and Critical Care Medicine, The Affiliated Changsha Central Hospital, Hengyang Medical School, University of South China, Changsha, China; ^2^Department of Cardiovascular Medicine, The Affiliated Changsha Central Hospital, Hengyang Medical School, University of South China, Changsha, China

**Keywords:** asthma, Life’s Essential 8, health behaviors, cardiovascular health, mortality

## Abstract

**Background:**

Research indicates that Life’s Essential 8 (LE8) has health-promoting effects for many diseases, yet few studies have explored its association with asthma patients. This research aimed to investigate the relationships between LE8 and all-cause mortality in asthma patients.

**Methods:**

We conducted a retrospective cohort analysis of seven cycles of data from the 2005–2018 National Health and Nutrition Examination Survey (NHANES). The impact of LE8, which includes four health behaviors (diet, physical activity, smoking, and sleep) and four health factors (BMI, lipids, blood glucose, and blood pressure), on asthma mortality was analyzed using multivariate Cox proportional hazards models. Dose–response relationships between these indicators and mortality were examined using restricted cubic spline (RCS) analysis. Subgroup analyses and interaction tests were performed to verify the stability of the results.

**Results:**

The study included 3,321 asthma patients aged 20 or older, with a median follow-up of 91.03 months, during which 331 patients died. Each one-unit increase in LE8 score was associated with a 1.4% reduction in all-cause mortality risk (HR = 0.986, 95% CI: 0.974–0.998; *p* < 0.001). Patients with scores ≥80 had a 58.8% lower mortality risk than those with scores <50 (HR = 0.412, 95% CI: 0.203–0.837, *p* = 0.014). Each one-point increase in health behavior score was linked to a 1.3% decrease in mortality risk (HR = 0.987, 95% CI: 0.982–0.992; *p* < 0.001). Participants with optimal health behaviors (scores ≥80) had a 53.8% lower mortality risk than those with poor scores (<50; HR = 0.462, 95% CI: 0.275–0.777; *p* = 0.004). RCS analysis revealed linear associations of LE8 and health behavior scores with mortality, while the relationship between health factor scores and mortality was non-linear, with mortality risk decreasing as scores increased above 80. Subgroup analyses showed stable associations between exposure variables and mortality, particularly strong protective associations in high-income groups.

**Conclusion:**

Optimized LE8, health behavior scores, and health factor scores above 80 are associated with reduced all-cause mortality risk in asthma patients, supporting ideal cardiovascular health as an intervention strategy to lower asthma mortality.

## Introduction

Asthma is a heterogeneous chronic airway inflammatory disease that imposes a significant burden on global public health (1). In 2019, approximately 300 million people worldwide were affected by asthma, resulting in about 461,070 deaths every year (2). While the age-standardized prevalence of asthma has decreased globally, its incidence and mortality rates are still on the rise in many regions (3–5). Asthma’s burden in terms of premature death or reduced quality of life remains a big concern (2).

In recent years, there has been a rising concern over the impact of lifestyle factors on the occurrence and prognosis of asthma. Research revealed that unhealthy lifestyle factors are linked to an increased risk of asthma in adults (6), whereas healthy lifestyles can significantly affect the mortality risk of asthma patients. The Oxidative Balance Score (OBS) evaluates both dietary antioxidants (e.g., vitamins C, E, and polyunsaturated fatty acids) and pro-oxidants (e.g., smoking and alcohol). Each quartile increase in OBS is associated with a 63% reduction in all-cause mortality risk among asthma patients (HR = 0.37) (7, 8), indicating that healthy eating habits may lower the risk of asthma death. A 20-year Danish cohort study showed that self-reported leisure-time physical activity is related to a nearly 50% decrease in all-cause mortality for asthma patients (9). Moreover, avoiding tobacco smoke exposure can lead to a 41% decrease in hospitalizations for severe asthma attacks, indirectly reducing deaths associated with acute attacks (10). Identifying multiple modifiable risk factors offers ways to reduce asthma exacerbations and improve prognosis. Obesity and metabolic abnormalities can further worsen asthma prognosis and increase mortality (11, 12). Comprehensive management of comorbidities like diabetes and hypertension can increase the five-year survival rate of elderly asthma patients by 18% (13). Currently, comprehensive assessment studies on the impact of these factors on the prognosis of asthma patients are still limited.

Cardiovascular health (CVH) is closely linked to modifiable behavioral and environmental factors, including tobacco use, passive smoke inhalation, sedentary lifestyles, and pollutant exposure (14). To promote CVH, the American Heart Association (AHA) introduced the ‘Life’s Essential 8’ (LE8) framework in 2022, which integrates eight core metrics targeting both health behaviors and clinical indicators (15). Lifestyle-related practices focused on diet, physical activity, tobacco avoidance, and sleep; and biomarker-based parameters involving body mass index (BMI), lipid metabolism, glycemic control, and vascular pressure regulation. Higher LE8 levels are associated with a lower risk of all causes and cardiovascular-related mortality in US adults, regardless of Cardiovascular disease (CVD) (16, 17). This association extends to a reduced risk of multiple chronic diseases, including chronic obstructive pulmonary disease (COPD), gallstones, metabolic syndrome, non-alcoholic fatty liver disease, and metabolic-associated steatohepatitis (18–23).

Emerging evidence suggests an inverse relationship between LE8 composite scores and asthma incidence. Analyses of National Health and Nutrition Examination Survey (NHANES) datasets further demonstrated that individuals with optimized LE8 profiles exhibit a statistically significant reduction in asthma risk (24). Moreover, United Kingdom Biobank research found that for every standard deviation increase in LE8, the risk of new-onset asthma in adults decreases by 17% (HR = 0.83) (25). Research indicates that changing health-related behaviors, such as quitting smoking, exercising regularly, and controlling weight can prevent CVD and lower asthma incidence by improving immune regulation and airway health.

Despite growing interest in LE8’s clinical relevance, its potential role in predicting mortality outcomes among individuals with asthma remains unexplored. To address this knowledge gap, we leverage NHANES data to comprehensively evaluate associations between LE8 composite metrics (including health behavior and health factor scores) and all-cause mortality risk in this population.

## Methods

### Research design

Administered by the United States (US) Centers for Disease Control and Prevention, the NHANES serves as a critical epidemiological resource for evaluating population-level health metrics and disease determinants. It systematically gathers nationally representative datasets spanning nutrition, clinical health parameters, and epidemiological trends through a multi-phase stratified probabilistic sampling framework. By enrolling participants from diverse age groups, genders, racial/ethnic categories, and socioeconomic strata across the US, the survey ensures robust generalizability of its findings to the broader population.

This study adopted a retrospective cohort study design. The study subjects were asthma patients (*n* = 9,979) from the 2005–2018 NHANES. We excluded the following related data: (1) < 20 years old (*n* = 4,342); (2) missing LE8 score (*n* = 1,772); (3) suffering cancer (*n* = 5); (4) missing follow-up data (*n* = 5); (5) missing values of other related variables (*n* = 534). Ultimately, 3,321 participants were included in the analysis.

### Ascertainment of deaths

Data on all-cause mortality during the follow-up period were collected, up to December 31, 2019.

### The concept of LE8

LE8 is a comprehensive CVH assessment tool developed by the AHA, aimed at measuring an individual’s health level through quantifying health behaviors and biological markers (16, 26, 27). Its core includes the following eight dimensions:

Dietary Quality: Emphasizes balanced nutrition. 2. Physical Activity: Assesses exercise frequency and intensity. 3. Nicotine Exposure: Includes smoking or secondhand smoke exposure. 4. Sleep Health: Focuses on sleep duration and quality. 5. Body Mass Index (BMI): Measures the ratio of weight to height. 6. Lipid Level: Assesses the level of non-high-density lipoprotein (HDL) cholesterol in the blood. 7. Blood Glucose Level: Measured by fasting blood glucose or hemoglobin A1c (HbA1c). 8. Blood Pressure: Assesses the state of vascular health. Each dimension is quantified through standardized scores (0–100 points), with the total score reflecting the overall CVD status. A higher score indicates better health levels (16, 27).

The AHA has documented the detailed scoring methods for each indicator (15). The LE8 scoring framework stratifies cardiovascular health status into three tiers: high CVH (80–100), moderate CVH (50–79), and low CVH (0–49), based on cumulative adherence to the eight defined health metrics.

Dietary patterns were quantitatively evaluated through the Healthy Eating Index (HEI)-2015 framework, which assigns scores based on adherence to dietary guidelines for nutrient adequacy and food group balance. Relevant studies have been published on how to calculate the HEI-2015 from NHANES data (18, 28). Supplementary Table S1 summarizes the components of the HEI-2015 and their scoring criteria. Information on physical activity, smoking, and sleep was collected via self-reported questionnaires. During the physical examination, trained professionals measured participants’ blood pressure, height, and weight. Body mass index (BMI) was derived using the standard formula of body weight (kg) divided by the square of standing height (m^2^). Venous blood specimens collected from participants underwent centralized laboratory processing to quantify metabolic biomarkers, including fasting lipid profiles, plasma glucose concentrations, and HbA1c levels.

### The definition of asthma

The diagnostic criteria for asthma was based on a standardized questionnaire: ‘Have you ever been diagnosed with asthma by a healthcare professional?’ (mcq010).

### Data collection

We collected baseline data, including demographic characteristics (gender, age, race), socioeconomic factors (education level, marital status, poverty income ratio (PIR)), comorbid conditions (chronic bronchitis, emphysema, cancer, and CVD), and laboratory indicators (white blood cell count (WBC), glomerular filtration rate (GFR)).

Age was divided into three stages: 20 to 39 years, 40 to 59 years, and 60 years and above (18). Race classification included non-Hispanic (NH) white, NH black, Mexican Americans, Hispanic, and other races. PIR was calculated by the ratio of family monthly income to the poverty line level and was divided into three groups: less than 1.3 (low income), 1.3 to 3.5 (middle income), and 3.5 and above (high income) (18).

Educational attainment was stratified into three tiers: (1) incomplete secondary education, (2) high school diploma or equivalent qualification, (3) undergraduate degree or higher academic achievement (23). Marital status was divided into never married, married or cohabiting, and widowed/divorced/separated. Alcohol use status was operationalized through the validated survey item: ‘Within the past 12 months, have you consumed 12 or more standard alcoholic drinks, regardless of beverage type’. Drinking status was divided into never drank, drank before, and currently drinks.

Data on cancer (has a doctor or other health professional ever told you that you had cancer or malignancy), chronic bronchitis (ever told you that you have chronic bronchitis), emphysema (ever told you that you have emphysema), and CVD (ever been told you had coronary heart disease, congestive heart failure, a heart attack (myocardial infarction), angina, or a stroke) were collected through questionnaires. Venous blood specimens were obtained through standardized phlebotomy protocols and subsequently transferred to certified laboratories for WBC enumeration. GFR was estimated via the Modification of Diet in Renal Disease formula (29).

### Statistical analysis

To address the complex stratified probabilistic sampling framework inherent to NHANES, all analytical models incorporated the designated survey weights (wtmec2yr), thereby preserving the dataset’s national-level generalizability. Descriptive statistics for categorical variables were expressed as frequency distributions with adjusted proportions, whereas continuous measures were reported as weighted arithmetic means accompanied by their standard errors. To explore the relationship between LE8 and its components with all-cause mortality in asthma patients, we employed multivariate Cox regression models. Three models were established: Model 1 with no covariate adjustment; Model 2 adjusted for age, gender, and race; and Model 3 further adjusted for education level, marital status, PIR, drinking history, chronic bronchitis, emphysema, cancer, CVD, WBC and GFR. The relationship between LE8 score, healthy behavior score, healthy factor score, and all-cause mortality in asthma was described using Kaplan–Meier Survival Analysis. The dose–response associations between LE8 composite scores (including healthy behavior and healthy factor scores) and all-cause mortality in asthma were modeled via restricted cubic spline (RCS). For non-monotonic relationships, segmented Cox proportional hazards models were applied to estimate differential hazard ratio (HR) above and below identified inflection thresholds. To validate result stability, we performed sensitivity analyses excluding patients with chronic bronchitis and emphysema and also carried out subgroup analyses and interaction tests. The analytical framework was executed within the R software (v4.2.2), with a statistical significance threshold of *p* < 0.05.

## Results

### Baseline characteristics

**Figure 1:**
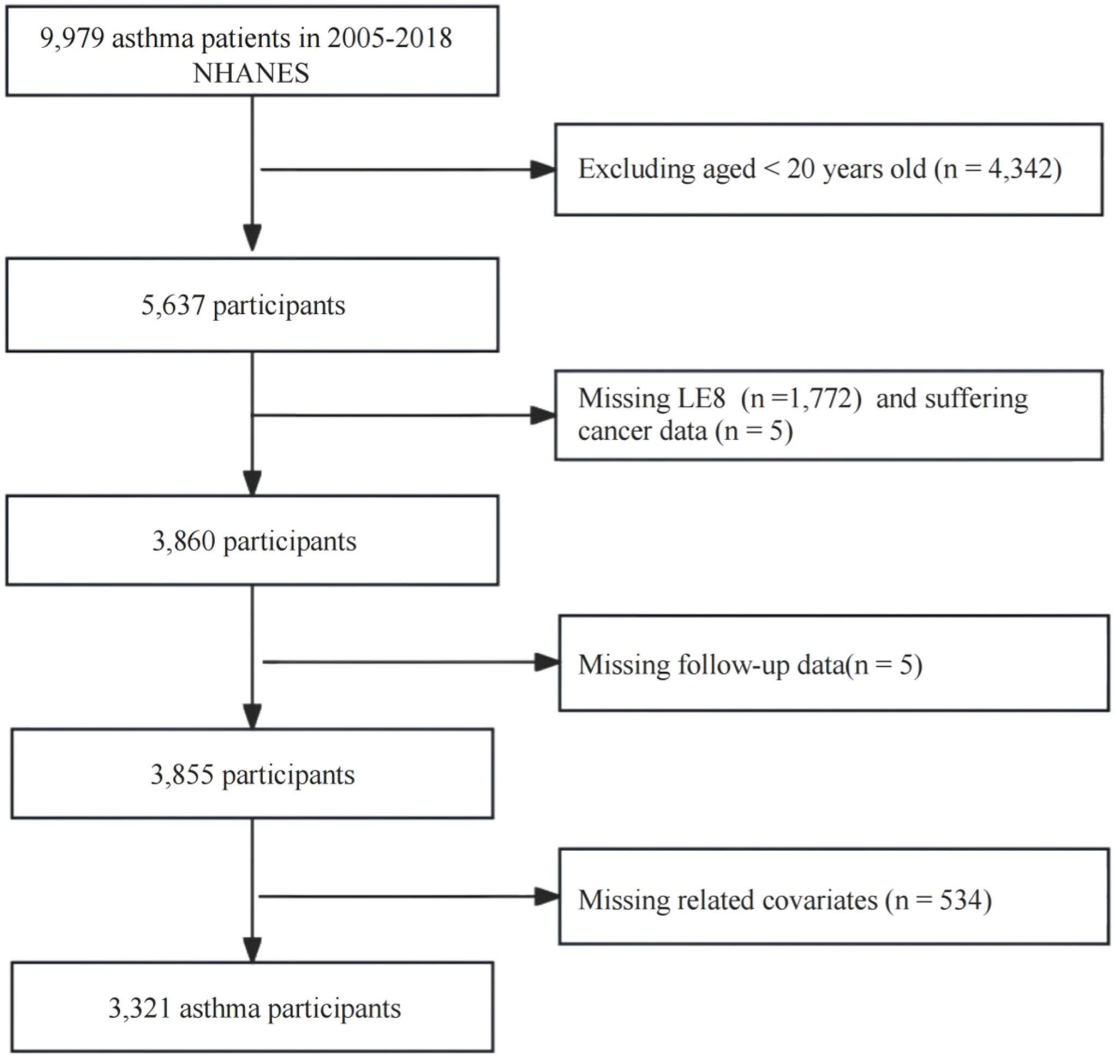
Flowchart of the standard for participants enrolled in the study.

A total of 3,321 adult asthma patients were included in the study. Participants were followed for an average of 91.03 months. The proportion of individuals aged ≥ 60 years was significantly higher in the mortality group (66.67%) compared to the survival group (20.15%; *p* < 0.0001). The mortality rate was significantly lower among those with a college degree or higher, with 51.78% in the mortality group versus 66.70% in the survival group (*p* < 0.0001). A higher mortality rate was observed among males, individuals who were widowed or divorced or separated, those with a low income (PIR < 1.3), and those with comorbid chronic diseases (chronic bronchitis, emphysema, cancer, and CVD). The survival group had significantly better scores in LE8, health behavior, and health factors compared to the mortality group (*p* < 0.0001). Detailed data are presented in Figure 1 and Table 1.

**Table 1:** Features of the study population, weighted.

Variables	Total (*n* = 3,321)	Alive (*n* = 2,990)	Deceased (*n* = 331)	*p*-value
Age (years), n (%)				< 0.0001
< 40	1,254(40.25)	1,234(42.66)	20(8.80)	
40–60	1,104 (36.29)	1,035 (37.19)	69 (24.53)	
≥ 60	963 (23.45)	721 (20.15)	242 (66.67)	
Gender, n (%)				0.02
Female	1897 (58.33)	1746 (58.95)	151 (50.32)	
Male	1,424 (41.67)	1,244 (41.05)	180 (49.68)	
Race, n (%)				0.002
Non-Hispanic White	1,656 (72.41)	1,443 (71.74)	213 (81.27)	
Non-Hispanic Black	770 (11.42)	702 (11.54)	68(9.92)	
Mexican American	309(4.83)	291 (5.05)	18 (2.04)	
Other Hispanic	291(4.77)	275 (4.97)	16 (2.07)	
Other Race	295(6.56)	279 (6.70)	16 (4.69)	
Education level, n (%)				< 0.0001
Less than high school	643 (12.53)	539 (11.73)	104 (22.95)	
High School or equivalent	734 (21.83)	650 (21.57)	84 (25.27)	
College Graduate or above	1944 (65.64)	1801 (66.70)	143 (51.78)	
Marital status, n (%)				< 0.0001
Never married	705 (19.90)	679 (20.76)	26(8.71)	
Married/Living with a partner	1812 (60.04)	1,654 (60.69)	158 (51.52)	
Widowed/Divorced/Separated	804 (20.06)	657 (18.55)	147 (39.77)	
PIR, n (%)				< 0.0001
< 1.3	1,169 (24.19)	1,023 (23.23)	146 (36.81)	
1.3–3.5	1,145 (33.39)	1,012 (32.88)	133 (40.08)	
≥ 3.5	1,007 (42.42)	955 (43.89)	52 (23.11)	
Alcohol consumption, n (%)				< 0.0001
Never	364(8.61)	327(8.40)	37 (11.41)	
Former	571 (14.34)	433 (12.49)	138 (38.52)	
Now	2,386 (77.05)	2,230 (79.11)	156 (50.07)	
Chronic bronchitis, n (%)				< 0.0001
No	2,641 (80.70)	2,431 (82.00)	210 (63.66)	
Yes	680 (19.30)	559 (18.00)	121 (36.34)	
Emphysema, n (%)				< 0.0001
No	3,117 (95.06)	2,885 (97.15)	232 (67.73)	
Yes	204(4.94)	105(2.85)	99 (32.27)	
Cancer, n (%)				< 0.0001
No	2,956 (88.08)	2,708 (89.27)	248 (72.51)	
Yes	365 (11.92)	282 (10.73)	83 (27.49)	
CVD, n (%)				< 0.0001
No	2,820 (88.18)	2,624 (90.50)	196 (57.82)	
Yes	501 (11.82)	366(9.50)	135 (42.18)	
WBC (1000cells/ul)	7.49 (0.05)	7.46 (0.06)	7.89 (0.16)	0.01
GFR	94.84 (0.55)	96.26 (0.55)	76.26 (1.67)	< 0.0001
Time (months)	91.03 (1.57)	93.13 (1.65)	63.61 (2.76)	< 0.0001
Life Essential 8 score	66.33 (0.44)	67.09 (0.45)	56.47 (1.28)	< 0.0001
Health behavior score	63.58 (0.63)	64.35 (0.65)	53.55 (1.43)	< 0.0001
Health factor score	69.08 (0.53)	69.82 (0.53)	59.39 (1.71)	< 0.0001

Continuous variables were expressed as weighted mean (standard error), categorical variables as counts (weighted percentage). n, numbers; PIR, poverty income ratio; WBC, white blood cells; GFR, glomerular filtration rate; CVD, Cardiovascular disease.

### LE8 score and asthma

Analyses presented in Table 2 revealed an inverse association between LE8 score and mortality risk. For each unit increase in LE8 score, the adjusted hazard of death decreased by 4.0% (HR = 0.960, 95% CI: 0.951–0.970; *p* < 0.001). This protective association persisted in fully adjusted models (Model II), with LE8 scores independently predicting a 1.4% reduction in mortality risk per unit increment (HR = 0.986, 95% CI: 0.974–0.998; *p* < 0.001). In terms of categorical comparison, the group with a score of ≥ 80 had a 58.8% reduction in the risk of death compared to the group with a score of < 50 (Model 2, *p* = 0.014), indicating a significant reduction in the risk of death among those with higher scores.

**Table 2:** Associations of Life’s Essential 8 score, health behavior score, and health factor score with all-cause mortality in patients of asthma.

	Crude model		Model 1		Model 2	
	HR (95% CI)	*p*-value	HR (95% CI)	*p*-value	HR (95% CI)	*p*-value
Life’s Essential 8 score						
Continuous variables	0.960 (0.951,0.970)	<0.0001	0.967 (0.958, 0.976)	<0.0001	0.986 (0.974,0.998)	0.027
Categorical variables						
< 50	ref		ref		ref	
50–80	0.373 (0.264,0.527)	<0.0001	0.439 (0.318, 0.605)	<0.0001	0.716 (0.500,1.024)	0.067
≥ 80	0.108 (0.060,0.194)	<0.0001	0.168 (0.090, 0.312)	<0.0001	0.412 (0.203,0.837)	0.014
*P* for trend		<0.0001		<0.0001		0.014
Health behavior score*						
Continuous variables	0.979 (0.973,0.986)	<0.0001	0.973(0.966, 0.980)	<0.0001	0.987 (0.980, 0.994)	<0.001
Categorical variables						
< 50	ref		ref		ref	
50–80	0.630 (0.461,0.860)	0.004	0.544(0.386, 0.767)	<0.001	0.840 (0.612, 1.152)	0.280
≥ 80	0.246 (0.160,0.376)	<0.0001	0.200(0.126, 0.317)	<0.0001	0.462 (0.275, 0.777)	0.004
*P* for trend		<0.0001		<0.0001		0.007
Health factor score#						
Continuous variables	0.975 (0.967,0.983)	<0.0001	0.989 (0.980, 0.997)	0.011	0.999 (0.991, 1.008)	0.878
Categorical variables						
< 50	ref		ref		ref	
50–80	0.660 (0.492,0.885)	0.006	0.856 (0.647, 1.133)	0.277	1.307 (0.977, 1.748)	0.072
≥ 80	0.278 (0.174,0.445)	<0.0001	0.697 (0.448, 1.083)	0.109	1.202 (0.767, 1.885)	0.422
*P* for trend		<0.0001		0.104		0.272

Crude model, variables unadjusted; Model 1, gender, age, and race were adjusted; Model 2, gender, age, race, education, marriage, poverty income ratio, alcohol consumption, chronic bronchitis, emphysema, cancer, white blood cells, cardiovascular disease, and glomerular filtration rate were adjusted. *Further adjusted health factor score. # further adjusted health behavior score. HR, hazard ratios; 95% CI, 95% confidence interval.

**Figure 2:**
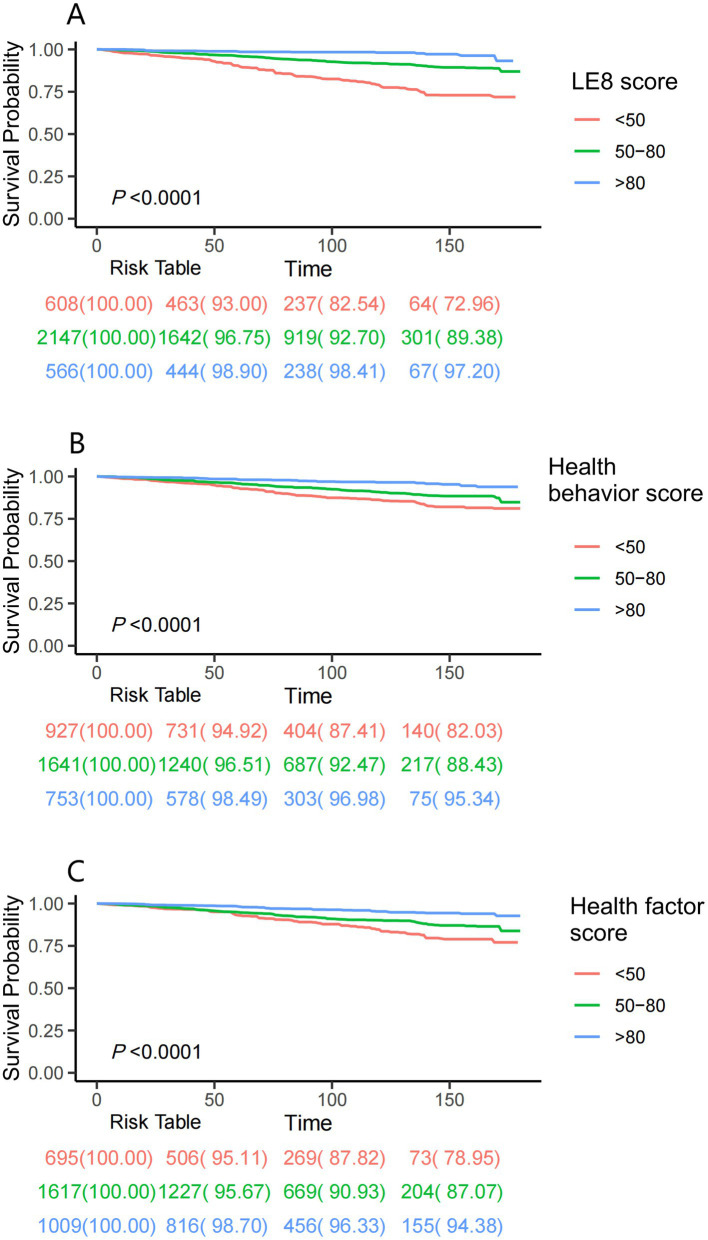
Kaplan–Meier survival analyses were used to assess the associations between LE8 score **(A)**, health behavior score **(B)**, health factor score **(C)**, and all-cause mortality in asthma patients. LE8: Life’s Essential 8.

**Figure 3:**
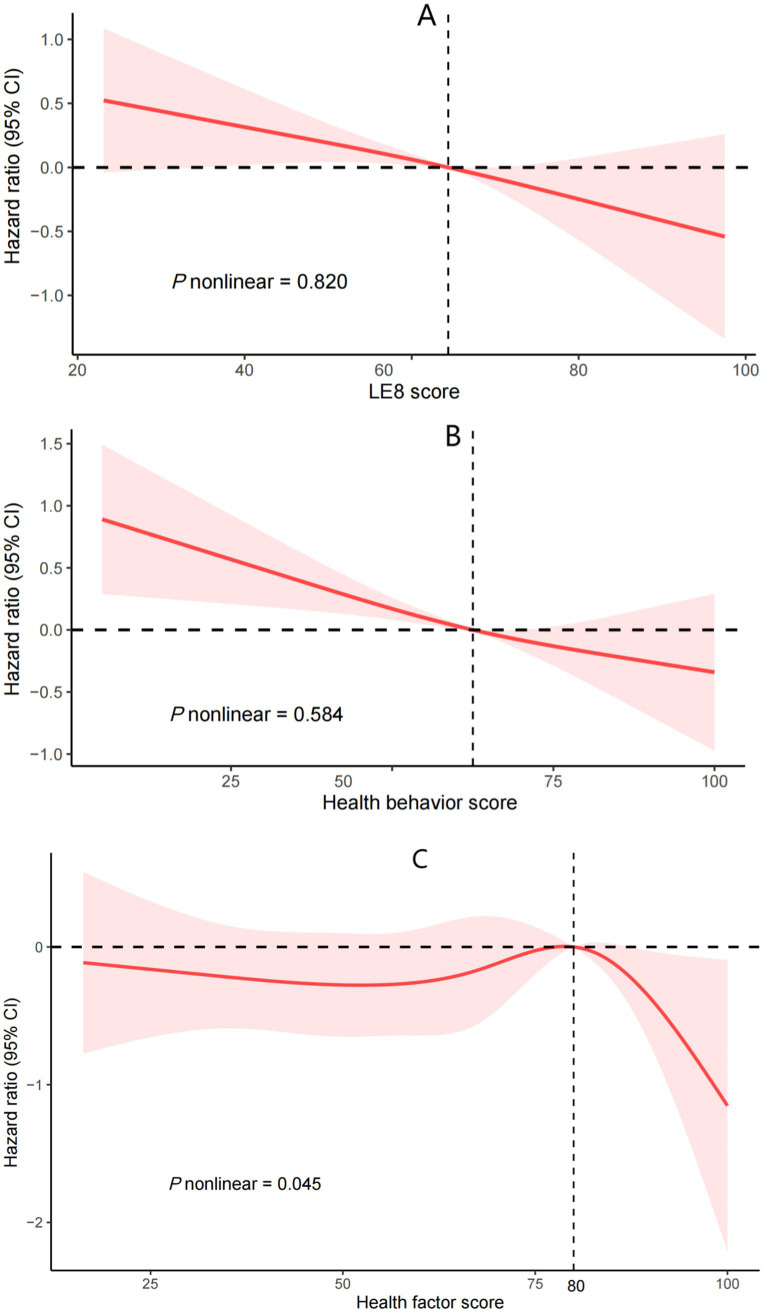
Cox regression using restricted cubic spline regression of LE8 score **(A)**, health behavior score **(B)**, health factor score **(C)**, with all-cause mortality. LE8: Life’s Essential 8. **(A)** was adjusted for gender, age, race, education, marriage, poverty income ratio, alcohol consumption, chronic bronchitis, emphysema, cancer, white blood cells, cardiovascular disease, and glomerular filtration rate. **(B)** was further adjusted for health factor score. **(C)** was further adjusted for health behavior score.

Kaplan–Meier survival analyses further illustrated that elevated LE8 composite score, health behavior score, and health factor score collectively corresponded to progressive declines in cumulative mortality incidence (Figure 2). Following full adjustment for covariates, RCS modeling confirmed a monotonic inverse relationship between LE8 scores and mortality risk in asthma cohorts (*P*-nonlinear = 0.820; Figure 3A).

### Health behavior score and asthma

In multivariable-adjusted analyses (Model 2), each incremental point in the health behavior score conferred a 1.3% reduced risk of death. (HR = 0.987, 95% CI: 0.982–0.992; *p* < 0.001). Categorical stratification further revealed a 53.8% mortality risk reduction among participants with optimal behavioral profiles (score ≥ 80) relative to those with suboptimal scores (< 50; *p* = 0.004). RCS analyses corroborated a monotonic dose–response relationship between health behavior scores and survival outcomes (*P*-nonlinear = 0.584; Figure 3B).

### Health factor score and asthma

Initial analyses (Crude and Model 1) demonstrated a statistically significant linkage between health factor metrics and mortality risk (*p* < 0.05). However, this relationship attenuated to non-significance upon full covariate adjustment in Model 2 (HR = 0.999, 95% CI: 0.988–1.010; *p* = 0.878). Restricted cubic spline modeling revealed a nonlinear dose–response pattern (*P*-nonlinear = 0.045; Figure 3C), with threshold analysis identifying 80 as the critical inflection point for mortality risk stratification. Below 80, it was unrelated to all - cause mortality (*p* = 0.88). Above 80, for every unit increase in the score, the risk of all-cause death increased by 13% (HR = 0.87;95% CI: 0.80, 0.95; *p* = 0.002; Table 3).

**Table 3:** Analysis of health factor score with all-cause mortality using piece-wise Cox regression.

Variable	HR (95% CI)	*p-*value
Infection point (K)		
The score < 80	1.00 (0.99, 1.01)	0.88
The score > 80	0.87 (0.80, 0.95)	0.002

Gender, age, race, education, marriage, poverty income ratio, alcohol consumption, chronic bronchitis, emphysema, cancer, white blood cells, cardiovascular disease, glomerular filtration rate, and health behavior score were adjusted. HR, hazard ratios; 95% CI, 95% confidence interval.

### Stratified analysis and interaction tests

**Figure 4:**
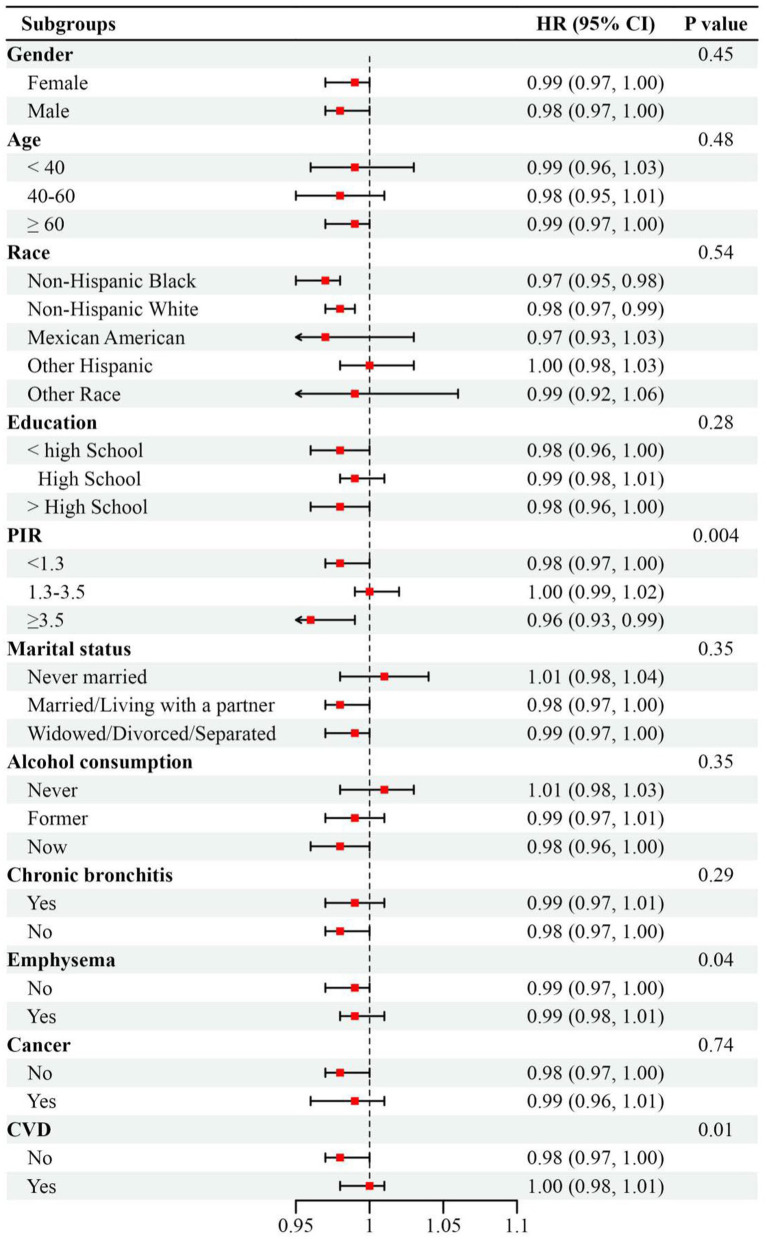
Subgroup analysis between Life’s Essential 8 score with all-cause mortality in patients of asthma, weighted. Adjusted by gender, age, race, education, marriage, poverty income ratio, alcohol consumption, chronic bronchitis, emphysema, cancer, white blood cells, cardiovascular disease, and glomerular filtration rate except for the stratification variable. Abbreviations: HR, hazard ratios; 95% CI, 95% confidence interval; PIR, poverty income ratio; CVD, Cardiovascular disease.

Subgroup analyses consistently demonstrated homogeneity in the inverse association between LE8 score and mortality risk across most demographic strata (*P* for interaction > 0.05). Notably, the mortality risk reduction attributable to LE8 optimization was most substantial among high-income participants (PIR ≥ 3.5; HR = 0.96, 95% CI: 0.93–0.99; *P* for interaction = 0.004). The impact of exposed variables on all-cause mortality was more pronounced in the non-emphysema or non-CVD subgroups (*P* for interaction < 0.05; Figure 4).

### Sensitivity analyses

We further excluded participants with chronic bronchitis (*n* = 680) and emphysema (*n* = 91). Sensitivity analyses showed similar inverse associations between LE8 score, health behavior score, health factor score, and all-cause mortality in asthma patients (Supplementary Tables 1, 2; Supplementary Figure 1). Stratified analyses also confirmed that the association between LE8 score and reduced all-cause mortality risk was more pronounced in high - income participants (Supplementary Table 3).

## Discussion

This study analyzed NHANES data and for the first time assessed the relationship between the LE8 comprehensive score, its components of health behaviors and health factors, and the all-cause mortality rate of asthma patients. Results showed that higher LE8 and health behavior score were significantly associated with reduced mortality risk. Specifically, compared to scores <50, higher LE8 scores, particularly those ≥80, were linked to a nearly 60% reduction in mortality risk. Subgroup analyses confirmed the robustness of these findings, revealing a stronger protective effect of LE8 scores in high-income individuals (*P* for interaction = 0.004). This suggests that limited access to healthcare resources and higher environmental exposure risks in economically disadvantaged groups may diminish the potential benefits of healthy behaviors.

Growing evidence suggests that the association between LE8 metrics and asthma pathogenesis may predominantly arise from shared etiological pathways, including chronic inflammatory states, systemic oxidative imbalance, and adiposity-related metabolic dysregulation, alongside modifiable behavioral and environmental determinants such as active/passive tobacco exposure, sedentary lifestyles, and exposure to environmental pollutants (14). Asthma is linked to higher levels of inflammation and oxidative stress (24, 30). Dietary patterns may influence inflammation, oxidative stress, mucus hypersecretion, and airway remodeling, with healthier eating habits associated with fewer asthma symptoms and better asthma control (31, 32). Tobacco smoke boosts asthma risk as its reactive oxygen species can trigger oxidative stress, worsening airway hyper-responsiveness in asthma (33–35). Smoking cessation can curb inflammation and oxidative stress (36). Higher physical activity levels are linked to better asthma control and reduced systemic inflammation markers like IL-6 and TNF-*α* (37–39). Conversely, unhealthy lifestyle factors such as poor diet, sedentary behavior, and obesity are associated with worse asthma control and reduced quality of life (40). The core features of metabolic syndrome, including obesity, insulin resistance, hypertension, and dyslipidemia, are closely related to chronic low-grade inflammation and oxidative stress. These conditions can worsen airway inflammation, leading to deteriorated asthma control and increased risks of exacerbations and mortality (14). These are potential health behavior and health factor indicators within LE8.

Previous studies have examined individual behavioral indicators’ links to asthma, whereas LE8 assesses multiple health behavior and factor indicators. Evidence showed LE8, health behavior score, and health factor score were negatively associated with asthma risk, with inflammation and oxidative stress partly mediating this link (24). This study revealed a linear inverse association between LE8, health factor score, and asthma mortality. However, health factor score exhibited a non-linear link to asthma mortality. Our findings align with Jiao Xu et al., who reported a nonlinear association between health factor scores and asthma prevalence, and a linear association with health behavior scores (41). Compared to previous studies on LE8-related mortality, in individuals with COPD, a 10-point increase in the LE8 score is associated with a 4% reduction in all-cause mortality (23). In patients with metabolic syndrome, LE8 and health behavior score shows linear associations with all-cause and CVD mortality, whereas health factor score exhibits a nonlinear relationship (22). This is consistent with the results of this study. Possible reasons include survivor bias, reverse causality, or overadjustment in participants. These results suggest that different LE8 levels might have diverse impacts on asthma. When the score exceeds 80, higher scores are linked to a lower risk of asthma-related death. The results highlight the importance of optimizing cardiovascular health metrics, enhancing healthy behaviors, and achieving high health factor score for better asthma prognosis. Subgroup analyses revealed that higher LE8 scores offer the strongest protective effect in the PIR > 3.5 group. This aligns with past research indicating that disadvantaged socioeconomic groups are more adversely affected by unhealthy lifestyles, and that low socioeconomic status plays a significant role in adult asthma progression (6, 42). These findings provide a theoretical basis for developing public health strategies to improve asthma patient prognoses. It is necessary to promote low-cost lifestyle interventions, such as community fitness programs, free smoking cessation services, and education on healthy eating and proper sleep. Moreover, poverty-reduction social policies are essential and must be implemented.

This study has several limitations. Residual confounding factors, such as unmeasured environmental, genetic factors, or the absence of asthma-specific variables, such as medication adherence, lung function, or exacerbation history, which may influence both LE8 components and mortality risk. The reliance on self-reported behaviors may introduce bias. The cross-sectional nature of the LE8 score fails to capture the dynamic changes in health behaviors. Nonetheless, this study offers new evidence on the all-cause mortality risk in asthma patients through a large sample size, multi-dimensional adjustments, and subgroup analyses. Future research could explore intervention trials testing LE8-guided lifestyle programs across diverse socioeconomic settings and further examine the health impacts of each LE8 component in asthma patients. Additionally, more research is needed to uncover the mechanisms behind the observed associations and develop targeted interventions based on these findings.

## Conclusion

Higher LE8, health behavior score, and health factor score are linked to lower all-cause mortality risk in asthma patients. Optimizing cardiovascular health, especially through modifiable behaviors, is an intervenable strategy to reduce mortality in adults with asthma.

## Data Availability

The datasets presented in this article are not readily available because authors can provide raw data if reasonably requested. Requests to access the datasets should be directed to Sue Zhao, 2018050785@usc.edu.cn.
