# Supplementary material for: Association of Life’s Essential 8 with all-cause mortality in asthma patients: evidence from NHANES 2005–2018
Source: Front Nutr. 2025 Jun 17;12:1603875. doi: 10.3389/fnut.2025.1603875 (PMC12209392; doi:10.3389/fnut.2025.1603875)
Supplement: Supplementary file 1 [file Data_Sheet_1.zip › Supplementary Data Sheet 1/Supplementary figure 1.docx]

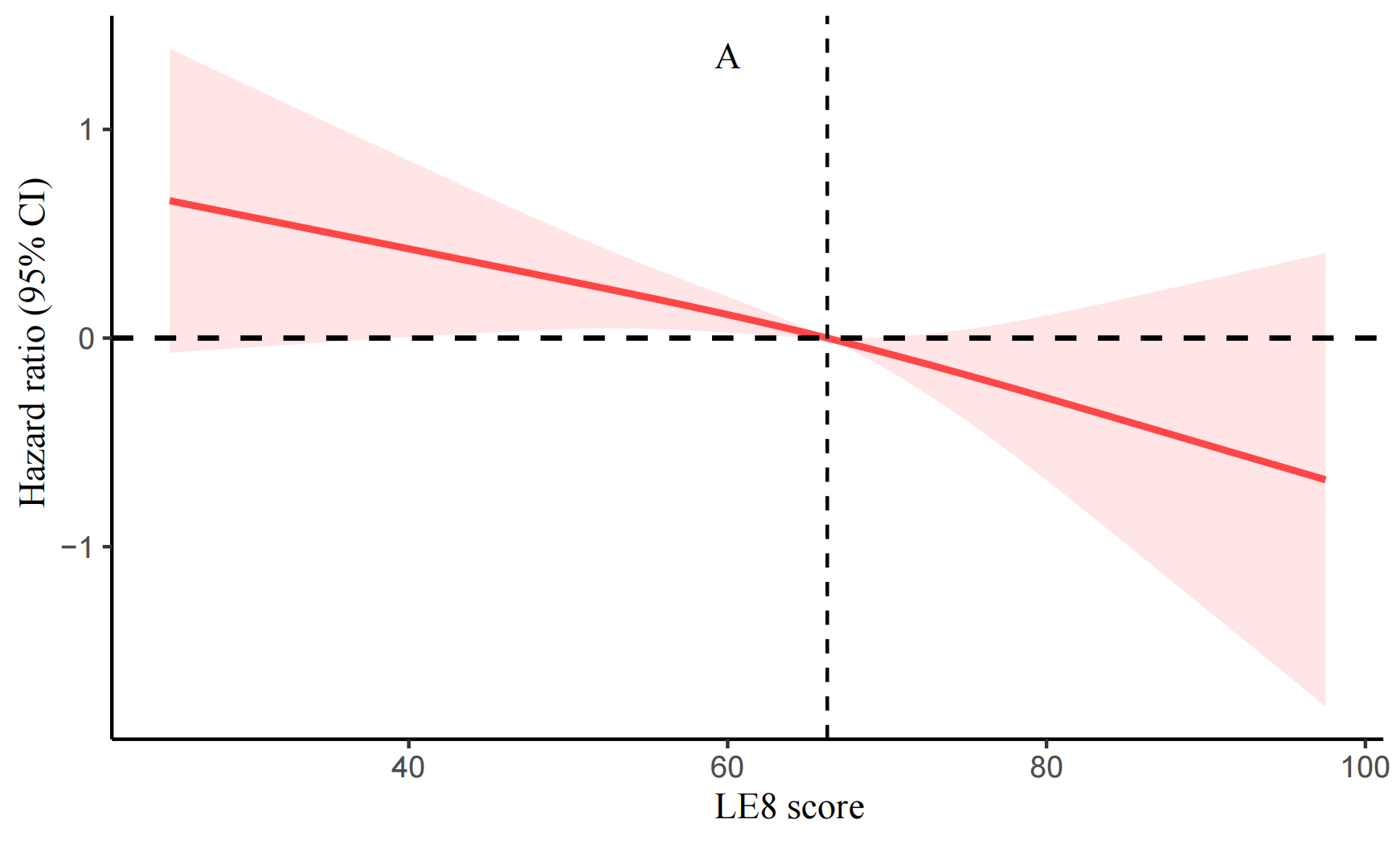


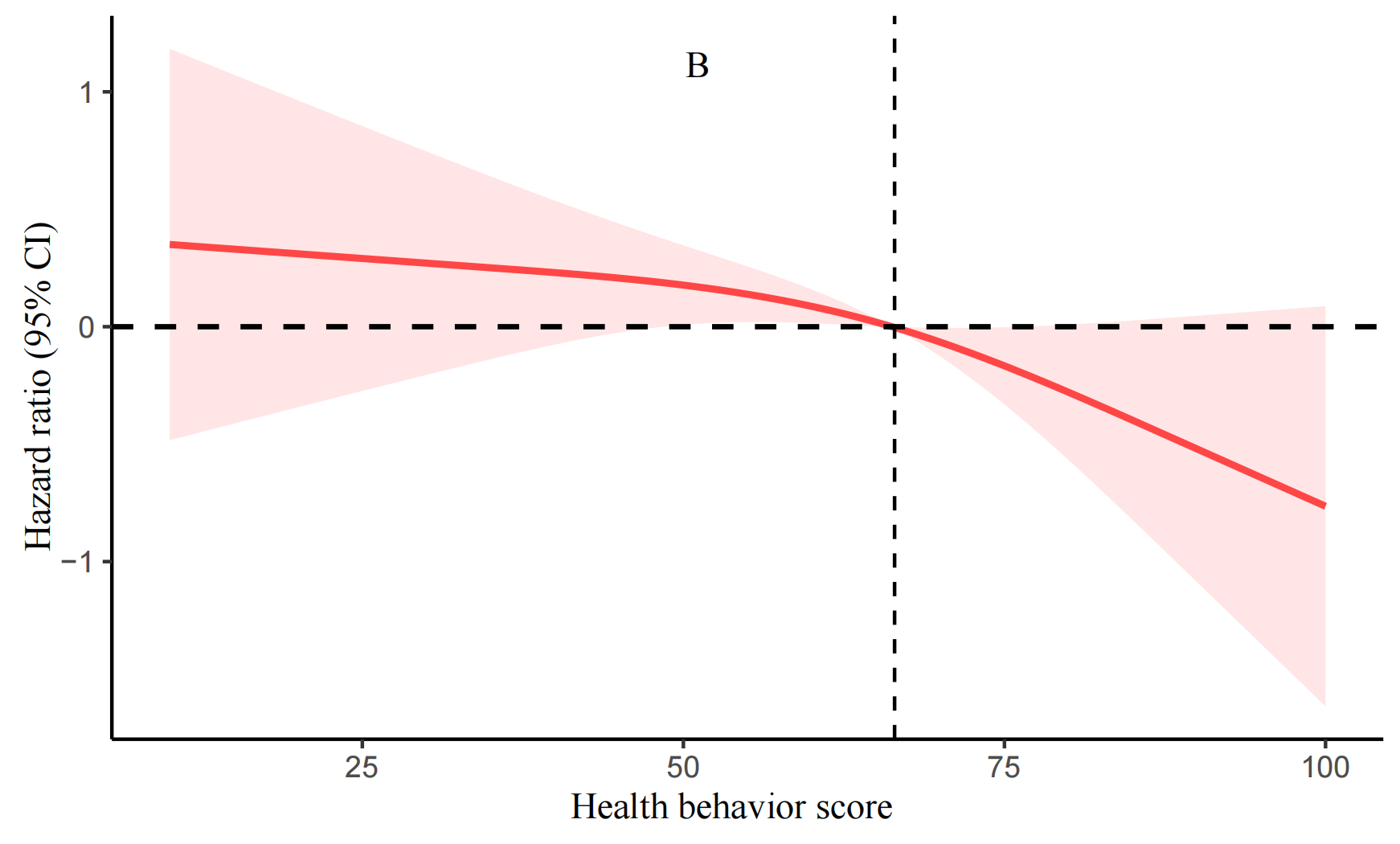


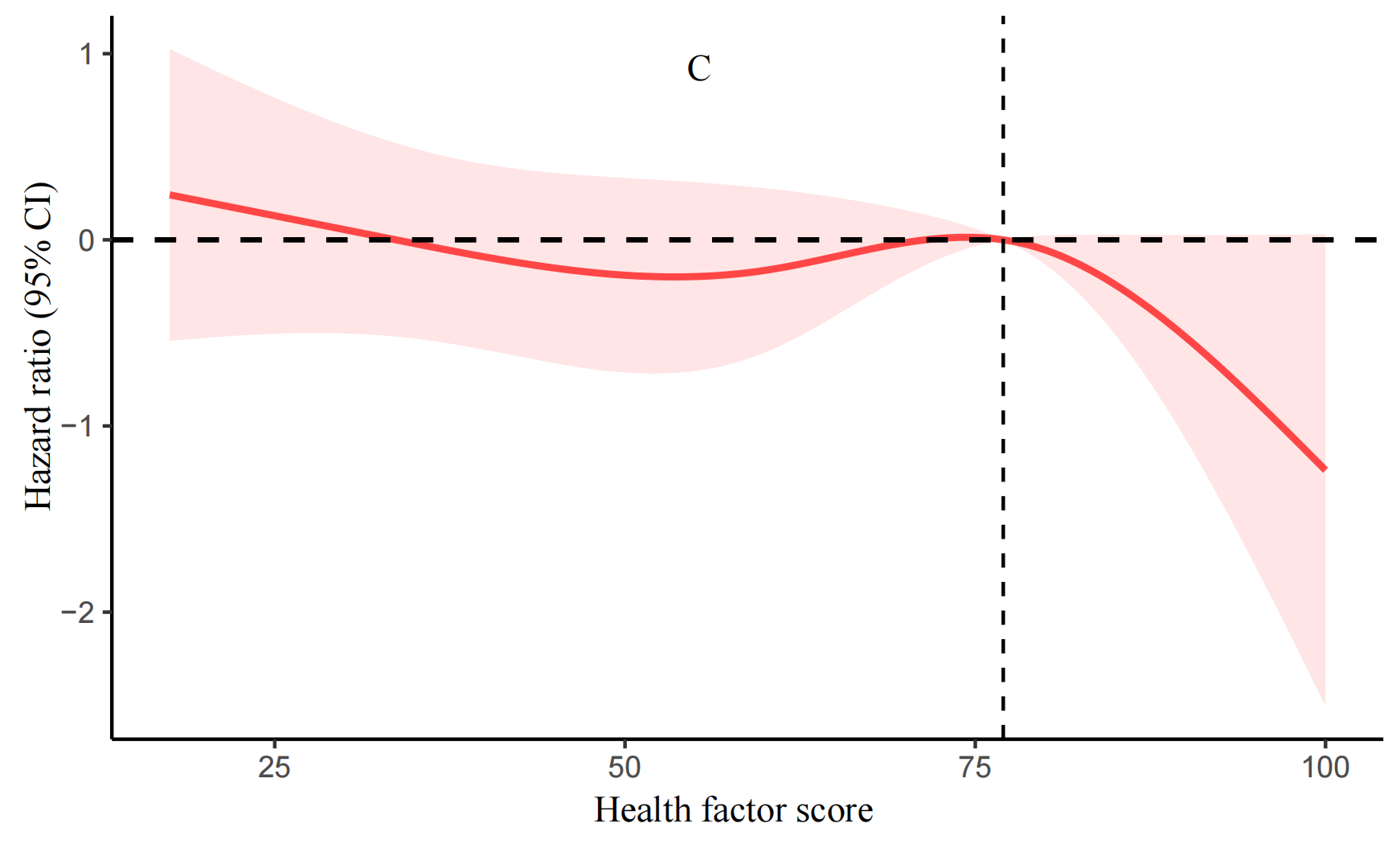


Supplementary figure 1 Cox regression using restricted cubic spline regression of LE8 score (A), health behavior score (B), health factor score (C), with all-cause mortality (n = 2550). LE8: Life’s Essential 8.

A was adjusted for gender, age, race, education, marriage, poverty income ratio, alcohol consumption, cancer, white blood cells, cardiovascular disease, and glomerular filtration rate.

B was further adjusted for health factor score.

C was further adjusted for health behavior score.
