# Supplementary material for: Association of Life’s Essential 8 with all-cause mortality in asthma patients: evidence from NHANES 2005–2018
Source: Front Nutr. 2025 Jun 17;12:1603875. doi: 10.3389/fnut.2025.1603875 (PMC12209392; doi:10.3389/fnut.2025.1603875)
Supplement: Supplementary file 1 [file Data_Sheet_1.zip › Supplementary Data Sheet 1/Supplementary Materials S1.docx]

## Healthy Eating Index-2015 components, point values, and standards for scoring

| **Component** | **Maximum points** | **Standard for maximum score** | **Standard for minimum score of zero** |
| --- | --- | --- | --- |
| **Adequacy** |  |  |  |
| Total Fruits | 5 | ≥0.8 c equivalents/1,000 kcal | No fruit |
| Whole Fruits | 5 | ≥0.4 c equivalents/1,000 kcal | No whole fruit |
| Total Vegetables | 5 | ≥1.1 c equivalents/1,000 kcal | No vegetables |
| Greens and Beans | 5 | ≥0.2 c equivalents/1,000 kcal | No dark green vegetables or beans and peas |
| Whole Grains | 10 | ≥1.5 oz equivalents/1,000 kcal | No whole grains |
| Dairy | 10 | ≥1.3 c equivalents/1,000 kcal | No dairy |
| Total Protein Foods | 5 | ≥2.5 oz equivalents/1,000 kcal | No protein foods |
| Seafood and Plant Proteins | 5 | ≥0.8 c equivalents/1,000 kcal | No seafood or plant proteins |
| Fatty Acids | 10 | (PUFAs^a^+MUFAs^b^)/SFA^c^ ≥2.5 | (PUFAs+MUFAs)/SFAs ≤1.2 |
| **Moderation** |  |  |  |
| Refined Grains | 10 | ≤1.8 oz equivalents/1,000 kcal | ≥4.3 oz equivalents/1,000 kcal |
| Sodium | 10 | ≤1.1 g/1,000 kcal | ≥2.0 g/1,000 kcal |
| Added Sugars | 10 | ≤6.5% of energy | ≥26% of energy |
| Saturated Fats | 10 | ≤8% of energy | ≥16% of energy |

^a^PUFAs = polyunsaturated fatty acids.
^b^MUFAs = monounsaturated fatty acids.
^c^SFAs = saturated fatty acids.
