# Supplementary material for: Association of Life’s Essential 8 with all-cause mortality in asthma patients: evidence from NHANES 2005–2018
Source: Front Nutr. 2025 Jun 17;12:1603875. doi: 10.3389/fnut.2025.1603875 (PMC12209392; doi:10.3389/fnut.2025.1603875)
Supplement: Supplementary file 1 [file Data_Sheet_1.zip › Supplementary Data Sheet 1/Supplementary table 1.docx]

| **Supplementary table 1** Associations of Life’s Essential 8 score, health behavior score, and health factor score with all-cause mortality in patients of asthma (n = 2550) | | | | | | | |
| --- | --- | --- | --- | --- | --- | --- | --- |
|  | **Crude model** |  | **Model 1** |  | **Model 2** | |  |
|  | **HR (95% CI)** | ***P*-value** | **HR (95% CI)** | ***P*-value** | **HR (95% CI)** | | ***P*-value** |
| **Life Essential 8 score** |  |  |  |  |  | |  |
| Continuous variables | **0.959(0.944,0.975)** | **<0.0001** | **0.968(0.955, 0.982)** | **<0.0001** | **0.983(0.966, 0.999)** | | **0.042** |
| Categorical variables |  |  |  |  |  | |  |
| < 50 | ref |  | ref |  | ref | |  |
| 50-80 | **0.360(0.215,0.603)** | **<0.001** | **0.438(0.263, 0.731)** | **0.002** | **0.603(0.355, 1.023)** | | **0.061** |
| ≥ 80 | **0.092(0.033,0.261)** | **<0.0001** | **0.142(0.053, 0.382)** | **<0.001** | **0.282(0.098, 0.812)** | | **0.019** |
| *P* for trend |  | **<0.0001** |  | **<0.0001** |  | | **0.008** |
| **Health behavior score*** |  |  |  |  |  | |  |
| Continuous variables | **0.983(0.974,0.992)** | **<0.001** | **0.977(0.968, 0.986)** | **<0.0001** | **0.989(0.980, 0.998)** | | **0.014** |
| Categorical variables |  |  |  |  |  | |  |
| < 50 | **ref** |  | **ref** |  | **ref** | |  |
| 50-80 | **0.866(0.564,1.328)** | **0.510** | **0.706(0.444, 1.122)** | **0.140** | **1.020(0.672, 1.548)** | | **0.925** |
| ≥ 80 | **0.279(0.145,0.537)** | **<0.001** | **0.219(0.115, 0.416)** | **<0.0001** | **0.402(0.200, 0.806)** | | **0.010** |
| *P* for trend |  | **<0.0001** |  | **<0.0001** |  | | **0.010** |
| **Health factor score#** |  |  |  |  |  | |  |
| Continuous variables | 0.970(0.958,0.982) | <0.0001 | 0.984(0.972, 0.997) | 0.015 | 0.994(0.983, 1.006) | | 0.315 |
| Categorical variables |  |  |  |  |  | |  |
| < 50 | ref |  | ref |  | ref | |  |
| 50-80 | 0.550(0.351,0.862) | 0.009 | 0.755(0.488, 1.166) | 0.205 | 1.063(0.695, 1.624) | | 0.779 |
| ≥ 80 | 0.208(0.096,0.448) | <0.0001 | 0.503(0.244, 1.037) | 0.063 | 0.842(0.423, 1.676) | | 0.625 |
| *P* for trend |  | <0.0001 |  | 0.054 |  | | 0.690 |
| Crude model, variables unadjusted; | | | | | | | |
| Model 1, gender, age, and race were adjusted; | | | | | | | |
| Model 2, gender, age, race, education, marriage, poverty income ratio, alcohol consumption, cancer, white blood cells, cardiovascular disease, and glomerular filtration rate were adjusted. | | | | | | | |
| * further adjusted health factor score  # further adjusted health behavior score | | | | | |  |  |
| Abbreviations: HR, hazard ratios; 95% CI, 95% confidence interval. | | | | | |  |  |
