# Supplementary material for: Association of Life’s Essential 8 with all-cause mortality in asthma patients: evidence from NHANES 2005–2018
Source: Front Nutr. 2025 Jun 17;12:1603875. doi: 10.3389/fnut.2025.1603875 (PMC12209392; doi:10.3389/fnut.2025.1603875)
Supplement: Supplementary file 1 [file Data_Sheet_1.zip › Supplementary Data Sheet 1/Supplementary table 2.docx]

Supplementary table 2 Analysis of health factor score with all-cause mortality using piece-wise Cox regression (n = 2550)

| Variable | HR (95% CI) | P-value |
| --- | --- | --- |
| Infection point (K) |  |  |
| the score < 68.25 | 0.99(0.97, 1.01) | 0.37 |
| the score > 68.25 | 0.96(0.93, 0.99) | 0.01 |

Gender, age, race, education, marriage, poverty income ratio, alcohol consumption, cancer, white blood cells, cardiovascular disease, glomerular filtration rate, and health behavior score were adjusted.

Abbreviations: HR, hazard ratios; 95% CI, 95% confidence interval.
