# Supplementary material for: Association of Life’s Essential 8 with all-cause mortality in asthma patients: evidence from NHANES 2005–2018
Source: Front Nutr. 2025 Jun 17;12:1603875. doi: 10.3389/fnut.2025.1603875 (PMC12209392; doi:10.3389/fnut.2025.1603875)
Supplement: Supplementary file 1 [file Data_Sheet_1.zip › Supplementary Data Sheet 1/Supplementary table 3.docx]

| Supplementary table 3 Subgroup analysis between Life's Essential 8 score with all-cause mortality in patients of asthma, weighted (n = 2550) | | |
| --- | --- | --- |
| Subgroups | HR (95% CI) | P-value |
| Gender |  | 0.10 |
| Female | 0.97(0.95, 0.99) |  |
| Male | 1.00(0.98, 1.02) |  |
| Age |  | 0.88 |
| < 40 | 0.99(0.96, 1.02) |  |
| 40-60 | 0.98(0.94, 1.02) |  |
| ≥ 60 | 0.98(0.96, 1.00) |  |
| Education |  | 0.22 |
| < high School | 0.97(0.94, 1.00) |  |
| High School | 0.99(0.97, 1.01) |  |
| > High School | 0.98(0.96, 1.00) |  |
| PIR |  | 0.04 |
| <1.3 | 0.99(0.97, 1.01) |  |
| 1.3-3.5 | 0.99(0.97, 1.01) |  |
| ≥3.5 | 0.96(0.93, 1.00) |  |
| Marital status |  | 0.35 |
| Never married | 1.00(0.97, 1.03) |  |
| Married/Living with a partner | 0.97(0.95, 1.00) |  |
| Widowed/Divorced/Separated | 0.99(0.96, 1.01) |  |
| Alcohol consumption |  | 0.61 |
| Never | 0.99(0.95, 1.03) |  |
| Former | 0.97(0.94, 1.01) |  |
| Now | 0.98(0.97, 1.00) |  |
| Cancer |  | 0.54 |
| No | 0.98(0.96, 1.00) |  |
| Yes | 0.97(0.95, 1.00) |  |
| CVD |  | 0.10 |
| No | 0.98(0.96, 1.00) |  |
| Yes | 1.00(0.98, 1.02) |  |

PIR, poverty income ratio; HR, hazard ratios; CVD, Cardiovascular disease; 95% CI, 95% confidence interval.
